# PDMS Bonding Technologies for Microfluidic Applications: A Review

**DOI:** 10.3390/bios11080292

**Published:** 2021-08-23

**Authors:** Alexandra Borók, Kristóf Laboda, Attila Bonyár

**Affiliations:** Department of Electronics Technology, Faculty of Electrical Engineering and Informatics, Budapest University of Technology and Economics, H-1111 Budapest, Hungary; borok@ett.bme.hu (A.B.); kristof.laboda@edu.bme.hu (K.L.)

**Keywords:** PDMS, polymers, bonding technologies, bond strength

## Abstract

This review summarizes and compares the available surface treatment and bonding techniques (e.g., corona triggered surface activation, oxygen plasma surface activation, chemical gluing, and mixed techniques) and quality/bond-strength testing methods (e.g., pulling test, shear test, peel test, leakage test) for bonding PDMS (polydimethylsiloxane) with other materials, such as PDMS, glass, silicon, PET (polyethylene terephthalate), PI (polyimide), PMMA (poly(methyl methacrylate)), PVC (polyvinyl chloride), PC (polycarbonate), COC (cyclic olefin copolymer), PS (polystyrene) and PEN (polyethylene naphthalate). The optimized process parameters for the best achievable bond strengths are collected for each substrate, and the advantages and disadvantages of each method are discussed in detail.

## 1. Introduction

Polydimethylsiloxane (PDMS), a silicon-based elastomer, is considered a fundamental building block in microfluidics due to its many desirable material properties (e.g., elasticity, optical transparency, biocompatibility, low autofluorescence, excellent thermal and chemical stability, etc.) [1,2,3,4]. Additionally, due to its convenient patterning technologies, such as replica molding or soft lithography, traditional fabrication methods centering on glass or silicon etching could be replaced with faster, less-expensive solutions [5,6,7]. Today, for a functional microfluidic device, microchannels are usually created in PDMS, then a chip is formed by closing them with a solid or flexible substrate (e.g., glass, silicon, or various polymers) [8,9,10,11]. This closing method is referred to as bonding technology since irreversible chemical bonds are formed between the activated or functionalized PDMS and substrate surfaces. Silicon-based materials (e.g., glass or silicon) can be relatively easily bonded to PDMS via simple surface activation, and thus these are the most widespread ways of sealing the microchannels.

However, in several cases, another type of substrate is required for a given application area. A common requirement could be the flexibility of the substrate. With the spreading of flexible electronics created by additive manufacturing [12,13] (e.g., electrodes on flexible polymer substrates, such as polyimide, created by 2D or 3D printing of nanomaterials [14]), functional elements such as sensors, biosensors, or microelectrode arrays can be integrated onto the surface of the polymer substrate [15,16]. Besides microfluidics, PDMS is also often used for the encapsulation of surface integrated electronics, or as protective coatings [17,18]. For complex Lab-on-a-Chip (LoC) or micro total analysis systems (Micro-TAS), PDMS is also often required to be bonded to other polymer-based materials, such as PET (polyethylene terephthalate), PI (polyimide), PMMA (poly(methyl methacrylate)), PVC (polyvinyl chloride), PC (polycarbonate), COC (cyclic olefin copolymer), PS (polystyrene) or PEN (polyethylene naphthalate) [19,20,21]. PDMS–polymer bonding is usually more complicated and requires the chemical functionalization of at least one bonded surface. These methods are called chemical gluing since chemical bonds between the applied molecular monolayers and surface functional groups provide a strong bond between the attached surfaces. In the past ten years, many techniques have been developed based on the combinations of surface activation, chemical gluing, and adhesive-based methods to bond PDMS with various substrates [22,23,24]. Since for most microfluidic applications a good quality bond with high strength is critical (especially where high-pressures or flow rates are applied), it is imperative to select the most appropriate bonding method for our target application. Additionally, the optimization of the technological process parameters is essential since, as demonstrated later, the quality of the bond greatly relies on it.

Our paper provides a review of these various technologies to bond PDMS with other surfaces. It pivots around three comprehensive tables, and the sections are structured accordingly. Table 1 and Section 2 compare the testing methods which are commonly used to quantify bond strengths. The differences between these methods should be understood in order to be able to evaluate and compare our bond quality with others. Table 2 and Table 3 compare the bonding methods for various substrates, which are discussed in detail in Section 3 and Section 4. While Table 2 focuses on the achievable bond strengths for a given substrate (based on the test method), Table 3 provides the optimized process parameters to reach these results. It is important to emphasize that both tables contain only the optimized process parameters. For bond quantification in a wider parameter range, please see the references. Our review is also content with focusing only on these aspects of PDMS-based microfluidic fabrication. Recent comprehensive reviews on the general application of PDMS for microfluidics [2], biomedicine [25], or fabrication technologies [5,6,7,26] can be recommended for those interested in other aspects.

## 2. Bond-Strength Testing Methods

Quality check of the prepared bond and the quantification of bond strength are important for all applications. Many different testing methods are used for this purpose; as shown in Table 1, 12 different functionally different techniques could be distinguished. Although a high bond strength—quantified by either tensile strength or shear strength—is a general requirement, many applications have special needs, e.g., long-term channel integrity [27,28], high working pressures inside the channel [29,30], or high inlet velocity [31,32,33]. Thus, understanding the differences between these testing methods and their provided information is essential for picking the right test for a given application. Comprehension of the testing methods is also crucial for interpreting the different bond strengths that characterize the bonding technologies.

### 2.1. Manual Peeling/Delamination Test

In this simple qualitative test, the bonded PDMS piece is forcibly removed from the substrate by hand or with the help of some basic tools like a scalpel. If the PDMS cannot be easily removed, or upon removal, the PDMS block tears/breaks (cohesive failure) instead of detaching cleanly from the substrate (adhesive failure), the formation of chemical bonds can be assumed. This test is the cheapest and easiest to implement: no special equipment or sample preparation is needed. On the other hand, the test does not yield any quantitative data, the results are quite subjective and have poor reproducibility. Usually, the main purpose of this test is to verify bond formation and/or filter out faulty workpieces before quantitative bond-strength testing with another method [19,20,21,34,35,36,37].

### 2.2. Tensile Strength Measurements

The quality of the prepared bond is best characterized by its tensile strength; thus, this is one of the most commonly used quantitative testing methods. Several different approaches exist to measure this property.

**Figure 1:**
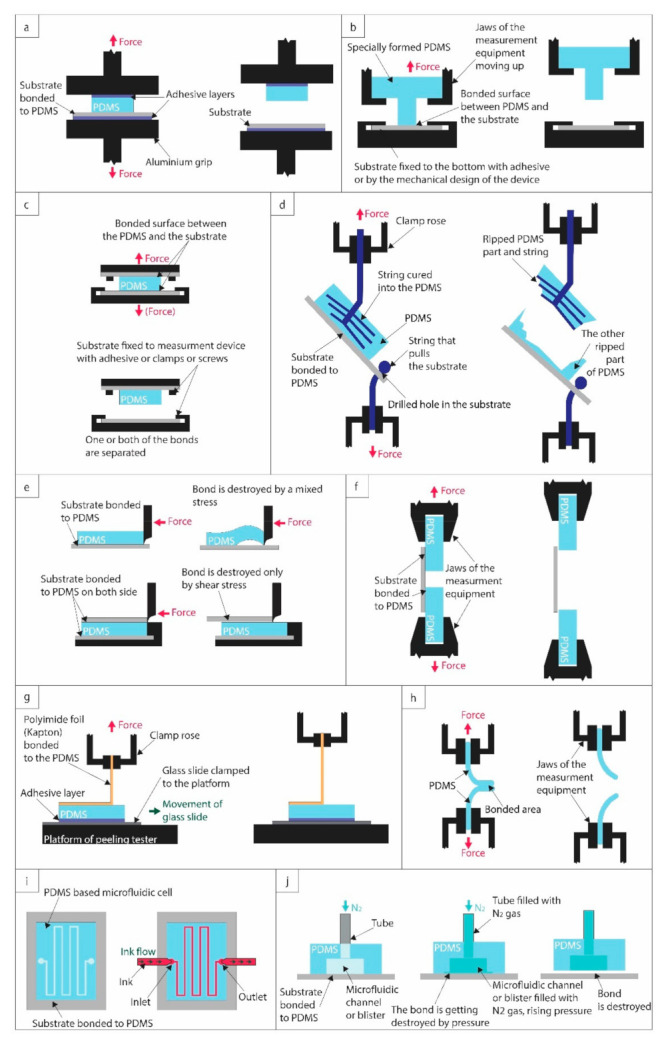
Illustrations of different bond-strength testing methods. (**a**) Tensile strength measurement with adhesive. (**b**) Cylinder-based tensile strength measurement with a specially designed PDMS piece. (**c**) Double-substrate bonding-based tensile strength measurement. (**d**) String-based tensile strength measurement. (**e**) Pushing-based shear strength measurement. (**f**) Pulling-based shear strength measurement. (**g**) Peeling test. (**h**) T-peeling test. (**i**) Leakage test. (**j**) Burst test.

With a standard tensile testing machine, grips can be used to pull the bonded materials apart from each other and measure the force required to detach the PDMS from the substrate. The general advantage of this test is that the experimental conditions are well defined, only normal forces act on the materials, and thus the results can be easily compared with other similarly executed measurements. The three approaches in Figure 1a–d differ in how the samples are fixed to the pulling grips. Although using adhesives to fix the upper (non-bonded) site of the PDMS block to the grip (or a specifically machined stump) might seem straightforward, this might cause inconvenience for some equipment. Additionally, the adhesive strength between the PDMS and the grip head (stump) must be higher than the characterized PDMS–substrate bond, otherwise, it will break prematurely (Figure 1a). Sunkara et al. used a silicone sealant (LC909N, Henkel, Germany) to bond the upper part of the PDMS to an aluminum jig, which held up to 490 kPa [38].

To avoid using adhesives, it is possible to create special jaws/grips to hold the PDMS and the substrate in the heads of the tensile testing machine. For this, the PDMS part should be explicitly molded for the purpose, e.g., in a cylinder shape (illustrated in Figure 1b, referred to as cylinder-based tensile strength measurement in Table 1). The protruding part of the cylinder is bonded to the substrate, while the flat part of the PDMS can be easily clamped to the equipment [39,40]. The substrate is similarly clamped to the bottom jaw. A disadvantage of this technique is that the used PDMS shape (which requires a separate molding form) is quite special and most possibly significantly differs from the design of the functional element, which cannot be directly used for this test. By contrast, the adhesive-assisted tensile testing could be performed even on the functional elements, e.g., bonded microfluidic cells, and thus combined with other testing methods (leakage test, burst test). The cylinder’s geometry should also be optimized: a too-long cylinder or a cylinder with a too-small base diameter can be torn down from the PDMS base. Too thin or fragile substrates (e.g., glass, silicon) are also vulnerable to breaking during the pulling.

The third solution is illustrated in Figure 1c. In this case, two layers of the substrate are bonded to both sides of the PDMS block. The substrate is fixed to the tensile strength testing machine with either clamps, screws, or adhesives [41,42]. The substrates should be thick enough to avoid breaking them during pulling. Additionally, if screws are used, holes are needed to be drilled into the substrates. The areas of the two bonds should be controlled precisely and taken into account upon the calculation of the tensile strength.

An alternative way for testing tensile strength is shown in Figure 1d. Here two twines are used to pull the PDMS/substrate assembly apart. One fixes the substrate to a pulling head via a drilled hole. A second twine is inserted into the PDMS pre-polymer and permanently cured into it. After bonding the PDMS with the substrates, the assembly is pulled apart. Although this pull test is performed in several works (e.g., by the team of Nae Yoon Lee [43,44,45,46,47]), it might have some drawbacks. Although cohesive failures can be easily distinguished from adhesive failures, the tensile strength cannot be adequately calculated in the latter case. Due to the nature of the assembly and the twine distribution inside the PDMS block, the pulling force cannot be considered perfectly normal and homogenously distributed along the bonded area. The bond between the twine and the PDMS is also crucial. The twine might be torn out from the block without detaching the PDMS from the substrate.

### 2.3. Shear Strength Measurement

Although shear forces might be a bit less relevant in microfluidic applications, in some instances, shear strength measurements are also performed on the bonded structures. In a classical shear testing machine, a force parallel to the bonding plane is applied with a given shear rate, as illustrated in Figure 1e. The shear force and displacement are measured, which can be used to calculate shear strength (failing load divided by the bond area) and shear strain [48].

A general problem with the shear test in Figure 1e is that the PDMS detaches gradually from the substrates, which requires monitoring the propagation of peeling to calculate the delamination area, as demonstrated in [49]. Pushing a partially peeled PDMS can also modify the direction of the acting forces, distorting the results.

To tackle this issue, Wang et al. bonded two rigid substrates to either side of the PDMS, and their shear tester pushed the upper substrate until the joint pair failed in one step. In this way, the shear strength and shear strain of the bonds could be characterized more conveniently and precisely [48].

An alternative approach could be using a tensile testing machine in shear mode, as illustrated in Figure 1f. Although in some works the results of these tests are referred to as tensile strength [50], due to the parallel forces, it is more appropriate to refer to them as lap shear strength, which is defined as the failure load divided by the bond area [51]. There are two ways to perform this test: either two PDMS blocks can be bonded to one substrate (as in [50]), or two substrates can be bonded to one PDMS block to avoid the difficulty of clamping PDMS. The bond areas should be precisely controlled and considered in lap shear strength calculation.

### 2.4. Peel Test

Peel tests are also frequently used to assess the bond quality of adhesive joints exposed to peel forces. Peel testing requires at least one flexible component, which refers to the ability of the adherend to bend through 90° without breaking or cracking [52]. For example, Hoang et al. used a Kapton foil bent at 90° angle, as illustrated in Figure 1g [53]. As the upper clamp roses and pulls the Kapton with a constant speed, at the same time, the lower platform shifts to maintain the 90°. Samples were glued onto glass slides using a silicone adhesive to prevent the PDMS from lifting during peeling. The resulting peel strength is calculated as the constant load per the bonded area’s width required to continue peeling the joint after initiation, determined from the flat portion of the force–extension curve [52,53].

Another method for peel strength measurement is the so-called T-peel test, illustrated in Figure 1h, which is also adopted by standard bodies (e.g., ISO 11339 or ASTM D 1876) [52]. A drawback of this method is that it requires both parts of the bonded structure to be flexible. It is most commonly used to detach two bonded PDMS blocks, as in [54] and [55], but a PDMS block bonded with a flexible foil could also be investigated this way. Overall, peel tests are harder to be performed correctly and could be done on a limited number of substrate types. The resulting peel strength is also a bit less relevant than the more widely used tensile strength.

### 2.5. Leakage Test

The most practical way to test the functionality of a microfluidic chip is leakage testing. In this family of methods (illustrated in Figure 1i), different approaches are used to test different qualities. In the simplest static leakage test (sometimes also referred to as durability test), a fluid is injected into the microchannels and kept there for a given amount of time, from the shortest test durations of minutes–hours [40,42] to even as long as months [20], and channel integrity is tested afterward. For these tests, no flow is applied on the fluid after injection (static conditions), and for extended tests, the ports are hermetically sealed. Besides colored water, other solutions are sometimes also used to test the long-term durability and swelling of PDMS (e.g., acids, i.e., 1M HCl [36], bases, i.e., 1 M NaOH [36], or organic solvents, i.e., tetrahydrofuran (THF) [43]).

By contrast, in the case of dynamic leakage tests, a constant flow is applied to evaluate the hydrodynamic stability of the device. Usually, the flow rate is gradually increased to a point where channel integrity fails. For the sake of better comparability between different structures/geometries, besides the absolute maximum flow rates (expressed in mL/min), the ratio of the liquid volume that flows through the channel within 1 min and the channel volume is also frequently given (expressed as flow-volume/channel-volume/min). Without a doubt, this is one of the most widespread methods for testing microchannel functionality [19,35,36,38,43,44,45,46,47,56,57].

### 2.6. Burst Test

Although technically this method could be considered to be a special type of leakage test, it is commonly referred to as burst pressure test (illustrated if Figure 1j). In this case, fluids or gases are introduced into either the microchannels or a blister specifically designed for this test. The pressure is slowly increased until the integrity of the investigated structure is compromised, either by delamination, leakage, or burst. This test requires appropriate instrumentation, including pressure control (pump and pressure sensor). Although usually the burst pressure level can be easily identified as a rapid drop in the measured pressure, sometimes the continuous optical monitoring of the structures might be needed to detect finer damage (e.g., slight leakage might happen before the burst of the cell). It has to be noted that—depending on the bonding protocol—there might be a huge difference between the burst pressures measured with air/nitrogen and water. For example, Pečar et al. measured a 5-fold difference between the burst pressures obtained with air and water, respectively [37], explained by the hydrolysis of covalent bonds [58]. (For this reason, in Table 2, the medium used for the test is also indicated.) Burst testing is probably the most used functional method to quantify the strength of the prepared bonds [21,36,37,38,39,40,41,46,47,48,57,59,60,61,62].

## 3. Bonding Methods

**Figure 2:**
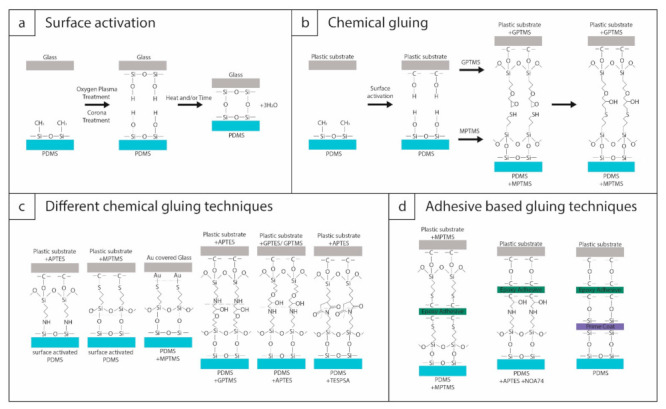
Illustrations of the process steps of (**a**) surface activation with oxygen plasma or corona treatment, (**b**) chemical gluing. (**c**) The most often used chemical gluing methods. (**d**) Adhesive-based or combined gluing methods.

In this section, the different strategies used to bond PDMS with various substrates are introduced in a general way. The specific protocols will be discussed in greater detail concerning the different substrate materials in Section 4. The general bonding strategies are illustrated in Figure 2. Silicon-based materials (e.g., glass or silicon) can be relatively easily bonded to PDMS. The process only requires surface activation (Figure 2a), which can be achieved either using corona discharge treatment [63,64] or oxygen plasma treatment on the surfaces [65,66,67]. For other substrate materials (e.g., thermoplastics), the bondable functional groups need to be created via surface functionalization. This method is often referred to as chemical gluing (Figure 2b,c). Sometimes these surface functionalization protocols are mixed with microscopic amounts of adhesives, as in Figure 2d.

### 3.1. Surface Activation by Oxygen Plasma Treatment

The general aim of surface activation is to remove contaminants and generate reactive chemical groups for covalent bonding. The convenient bonding of silicon-based materials through silanol groups (–Si–OH) is well-known in MEMS technologies (e.g., silicon wafer-level bonding) [68]. Through surface activation, the terminal methyl groups (–CH_3_) in PDMS (generally comprised of repeating units of –O–Si(CH_3_)_2_–) can be replaced by silanol groups. These can form covalent siloxane bonds (Si-O-Si) with a similar silanol group on another activated surface after the loss of a water molecule (Figure 2a) [59]. The processes taking place upon plasma/corona exposure can be quite complex since the material is simultaneously subjected to a mixture of energetic particles and radiation, e.g., electrons, ions, UV, and ozone, which can cause at least 12 different interactions (for details see [69]). The surface oxidation of PDMS and the increased concentration of hydroxyl groups can also lead to the formation of strong intermolecular bonds [69,70]. These reactions may change the surface properties significantly [71], either by the formation of a thin SiO_x_ layer, by increased cross-linking, or on the contrary, by the degradation of the network structure—all depending on the experimental conditions (e.g., plasma power, exposure time, etc.). Most of the proposed interactions upon plasma/corona exposure make the surface hydrophilic [69,72], and thus the quality of surface activation is generally monitored by water drop tests (contact-angle measurements). Exposed to normal ambient conditions, the treated surfaces quickly regain their hydrophobicity, mostly due to the migration of low molar mass chains from the bulk to the surface, the reorientation of polar groups on the surface into the bulk, or simply due to contamination [69,73,74]. Mechanical deformation (even compressive strains of less than 1%) were confirmed to speed up the recovery process [73], and thus treated PDMS samples should be handled with great care during alignment. Overtreated PDMS was also found to regain hydrophobicity faster, emphasizing the optimization of experimental conditions [73].

Plasma treatment is by far the most commonly used method for PDMS surface modification [74]. A large variety of gases can be used (e.g., oxygen, argon, nitrogen, hydrogen, fluorinate, etc.) from medium vacuum to atmospheric pressure for different purposes [75,76]. To create a silanol rich surface for PDMS bonding, oxygen plasma is used with pressures in the low–middle vacuum range. Besides gas content and pressure, the two other main process parameters are the plasma power and exposure time, which need to be optimized for successful bonding [20,49,54,55]. A significant advantage of plasma treatment is that with a common plasma chamber (reactor), these conditions can be fine-tuned and controlled precisely, yielding reproducible results and overall better bond quality. Low-pressure plasma is also less harmful to functional elements (e.g., thin-film metallic sensors) that might be integrated/embedded onto the treated surface, compared to corona treatment. Oxygen plasma surface activation is also frequently used as the first step in chemical gluing procedures [41,46].

### 3.2. Surface Activation by Corona Treatment

A corona treater is a device that generates a high voltage across an electrode at the tip of the unit. This handheld device is usually supplied with electrodes in different shapes for various applications [77,78]. The high potential of the electrode ionizes the surrounding air, creating a localized plasma called corona discharge. Corona treatment is a simple, cheap, and fast method which does not require expensive equipment or a special environment (the discharge can be generated at room temperature and atmospheric pressure, without the need for a vacuum system); it is portable, safe, and easy to be used [47,60,79]. A disadvantage of the technique is that it is usually performed manually. Although the discharge can be expanded to cover larger areas (with appropriate electrodes), the sample-electrode distance, movement speed, treatment time are all factors that need to be precisely controlled [19]. It has to be noted that the discharge could be harmful to metallic structures, e.g., it has been reported to have damaged a 35 nm thick plasmonic gold thin film on glass beyond applicability [50]. Corona surface activation can also be used combined with chemical gluing [43,47].

### 3.3. Surface Activation by UV/Ozone Treatment

The third possibility for enriching the PDMS surface with silanol groups is UV/ozone treatment [80,81]. Although this technique is significantly slower compared to both plasma and corona treatment, due to the greater penetration of photons, deeper surface modification (i.e., several 10 µm) could be achieved without any surface damage [74,82]. The process parameters (UV line, lamp power, exposure time) should be precisely selected to control the surface properties and hydrophobicity recovery [83,84]. The formation of a glassy, brittle SiO_x_ layer [85] might not be desirable for some applications that rely on the flexibility of PDMS and a matching substrate. UV/ozone is also used for surface activation in chemical gluing [50].

### 3.4. Chemical Gluing

As discussed before, PDMS can be conveniently bonded to glass or other silicon-based materials through siloxane bonds. To bond PDMS with other materials, e.g., thermoplastics, functional groups should be created either only on the target substrate or on both materials. These techniques are often referred to as chemical gluing, where molecular monolayers (acting as coupling agents) are anchored on the surfaces with specific terminal functional groups [36,45,86,87,88]. The most commonly used molecules are all organosilanes, namely APTES ((3-aminopropyl)triethoxysilane), MPTMS ((3-mercaptopropyl)trimethoxysilane), GPTES ((3-glycidyloxypropyl)triethoxysilane), GPTMS ((3-glycidyloxypropyl)trimethoxysilane), and TESPSA ((3-triethoxysilyl)propylsuccinic anhydride). Figure 2c illustrates the different bonding schemes between PDMS and a general polymer substrate by using these functional molecular layers. As can be seen, the silanol groups of the PDMS can be directly used to form bonds with the amino groups (–NH_2_) of APTES or the thiol groups (–SH) of MPTMS functionalized polymers. APTES can also be used paired with GPTES, GPTMS, or TESPSA, depending on the substrate, while MPTMS is usually paired with GPTMS.

During surface activation, the carbon backbones of thermoplastic substrates (e.g., PMMA or PC) are broken with corona or oxygen plasma treatment. These free carbons react with inorganic silicons of the silane coupling agent, which is used to functionalize substrate surfaces to form Si–O–C bonds [89]. It is important to emphasize that the process parameters of chemical gluing (e.g., concentration and temperature of the solution, thorough rinsing, drying, and subsequent thermal treatment to stabilize the self-assembled layers before bonding) need to be controlled for optimal results, similarly to the surface activation [41]. The hydrolytic stability of Si–O–C bonds and various chemical gluing protocols were also investigated in detail [89]. The exact protocols and achievable bonding strengths are given in Table 2 and Table 3, and discussed in the next section.

### 3.5. Adhesive-Based Gluing

Adhesive-based gluing—when a microscopic amount of adhesive materials is applied between the two surfaces instead of molecular layers—is usually not considered bonding. Depending on the target substrate, applying epoxy or silicone-based adhesives might prove significantly weaker than chemical gluing methods [89]. However, since adhesives are sometimes used in combination with chemical gluing methods to improve bond quality, these hybrid techniques are worth mentioning. A few examples are given in Figure 2d, using different types of epoxy adhesives (e.g., Norland Optical Adhesive (NOA74) [61], LePage Gel epoxy adhesive [53], Biocompatible Epoxy 301-2 [57]). MPTMS [53] and APTES [61] can both be used to covalently bind the epoxy adhesive layer (with thickness in a few µm range) with the PDMS. In a different technique, a silicone-based adhesive (PrimeCoat, ~1 µm) and an epoxy adhesive (3–4 µm) were spin-coated on top of the substrate [57].

## 4. Bonding Strategies for Various Substrates

In this section, the exact protocols used to bond PDMS with various substrates will be discussed in detail. Table 2 collects the applicable protocols for each substrate type and presents the achievable bonding strength, systematically ordered along with the testing methods of Table 1. Table 3 gives each protocol’s optimized process parameters to achieve the best results for a given substrate type.

### 4.1. PDMS

One of the most straightforward approaches to close a PDMS microchannel with a substrate is PDMS–PDMS bonding since a simple surface activation is sufficient, which can be performed in many ways. The most comprehensive studies on the optimization of oxygen plasma and corona treatment process parameters for the best achievable bonding strengths were performed by Bhattacharya et al. [59,60]. They mapped a wide range of RIE (Reacting Ion Etching) power (5–150 W), chamber pressure (20–1000 mTorr), and time of exposure (5–60 s) for both an inductively coupled high-density (ICP) plasma system, and a plasma-enhanced chemical vapor deposition (PECVD) system. The optimal parameters were found to be 700 mTorr, 20 W, and 30 s exposure, with an ICP system, which resulted in 400 kPa (58 psi) burst pressure [59]. In a newer work, they managed to reproduce these results with the same experimental conditions, also demonstrating huge variation (average burst pressure: 300 kPa, range: 180–715 kPa), and obtaining slightly smaller values with corona treated surfaces (average: 290 kPa, range: 227–380 kPa) [60]. The better reproducibility of corona treatment (handheld discharge unit, 15 kV output voltage for 30 s) is admittedly against expectations. It has to be noted that these burst pressures, obtained with optimized process parameters, are 2–3× better compared to other similar available data, e.g., Yousuff et al., who measured 105–180 kPa after oxygen plasma, and 87–95 kPa for UV/ozone treatment, respectively [62]. However, as also shown in Table 3, in many of these cases, the exact process parameters were not elaborated in the papers [45,62]. The parameters of atmospheric RF glow discharge plasma were optimized by Jung et al. [55], and similarly good results—in terms of peel test—were demonstrated with low-pressure RF air plasma as well [54].

As for chemical gluing, Lee et al. demonstrated a 2× increase in tensile strength (from 91 to 184 kPa) when using APTES/GPTMS gluing compared to normal oxygen plasma activation—although the plasma parameters are not given and presumably were not optimized either [45].

Another important technique that needs to be noted here is pre-polymer gluing (also referred to as ‘uncured PDMS adhesive’ or ‘partially cured adhesive’). In this approach, uncured/partially cured PDMS is placed between two fully cured substrates, or one of the two bonded pieces is only partially cured before making contact with the other substrate. Here, the pre-polymer adhesive or partially cured piece is cured during/after the bonding process. As an example, for partial cure bonding, Bhattacharya et al. pre-cured PDMS at 60 °C for 35 min before bonding and then allowed the piece to fully cure overnight following the bonding [60]. For both groups who tested this approach, the measured maximum burst pressures were at least 2× higher, compared to the same structures bonded with only either oxygen plasma or corona treatment (e.g., an average of 671 kPa), making it superior compared to other techniques [60,62]. Similarly good results were reported by Cao et al. with DMPMS-based (dimethyl-methylphenylmethoxy siloxane) pre-polymer gluing [35].

### 4.2. Glass (Silicon)

PDMS–glass and PDMS–silicon are also regularly used pairs to close PDMS microchannels. In the latter case, PDMS is bonded to the native oxide of silicon, so although glass is more frequently used, the protocols for bonding PDMS to silicon can be considered to be the same.

All of the approaches listed in Table 2 are based on surface activation similar to PDMS–PDMS bonding. The optimized oxygen plasma parameters with an ICP equipment were found to be 1000 mTorr chamber pressure, 20 W plasma power, and 30 sec exposure time [59]. The resulting 510 kPa (74 psi) burst pressure is more than 25% higher compared to PDMS–PDMS bonds also prepared with optimized oxygen plasma parameters [59]. For identical process parameters, others also reported significantly higher bonds between PDMS–glass than PDMS–PDMS, and the difference was even higher for UV/ozone treatment (95 to 314 kPa, respectively) [62]. Prepolymer gluing was also successfully used with glass substrates [35,62].

In Table 2, two application-specific examples are also given. Bonding PDMS to gold-coated glass [90] could be important for several sensor applications (either electrochemical or optical). For this purpose, surface activation + MPTMS functionalization of the PDMS could be used, relying on the terminal thiol groups to bond covalently to the gold surface. Bakouche et al. successfully demonstrated this method with an SPR (surface plasmon resonance) chip and even obtained higher lap shear strength values than for PDMS–glass bonding with simple corona surface activation [50]. Parylene, an organic polymer with a para-xylylene backbone, has many favorable properties considering microfabrication or microfluidics [91]. To irreversibly bond parylene with PDMS, Rezai et al. tried both pre-polymer gluing and plasma treatment with success [39]. By optimizing the process parameters of the latter (using a mixture of SF_6_ and N_2_), they obtained a very high bond tensile strength of 1.4 MPa and 145 kPa burst pressure (tested with water) [39].

### 4.3. PMMA

Similar to PDMS, poly(methylmethacrylate) (PMMA) is also a popular material for micro-TAS or LoC fabrication [92]. PMMA is an optically transparent thermoplastic (one of the least hydrophobic materials used in microfluidics), and its low price and compatibility with several microfabrication methods (e.g., injection molding, milling, hot embossing, etc.) makes it an ideal choice for disposable microfluidic devices. Since its direct bonding with PDMS is not possible [93] with an adequate bonding strength (e.g., with pre-polymer gluing, only 15 kPa tensile strength could be obtained [42]), a chemical gluing method has to be selected. There are numerous possibilities: nearly all of the illustrated approaches presented in Figure 2b,c can be used for PDMS–PMMA bonding, combined with either oxygen plasma or corona surface activation. Most of the protocols listed in Table 2 uses only one molecular adhesive. In these cases, the PMMA is functionalized with either APTES [37,38,41] or MPTMS [43] then bonded to a surface-activated PDMS. Others functionalize both surfaces with APTES/GPTMS [44] or APTES/TESPSA [46] pairs. By comparing the resulting tensile strength and burst pressure values, no direct improvement can be observed in favor of the double functionalization. With optimized plasma treatment and APTES coating parameters, high tensile strength (1.6 MPa [41]) and high burst pressures (>500 kPa, measured in air [37]) were obtained by coating only the PMMA piece.

An interesting approach is presented by Zhang et al., who used APTES functionalization on PMMA, followed by the addition of monoglycidyl ether terminated, low-molecular-weight PDMS [47]. In this way, PDMS-like terminals were placed onto the substrate (via the amine–epoxy bond), which can be subsequently bonded with bulk PDMS through classical siloxane bond formation. The aminosilane + monoglycidyl ether terminated PDMS combined functionalization thus leads back to PDMS–PDMS bonding, which can be used with a variety of substrates (e.g., PS, PC), with good results (580–620 kPa burst pressures) [47].

### 4.4. PC

Similar to PMMA, PC is another optically transparent thermoplastic with a high glass transition temperature (145 °C), low moisture absorption, and durability that makes it ideal for high-temperature applications. All of the chemical gluing protocols previously discussed for PMMA-PDMS bonding can be used for polycarbonate (PC) as well, including surface activation and APTES [37,38,58] or MTPMS [43] treatment, using APTES+ monoglycidyl ether terminated, low-molecular-weight PDMS functionalization [47], or using APTES/TESPSA [46] or APTES/ GPTMS [44] linker pairs. A notable difference is that with the identical bonding techniques, the resulting bond tensile strength or burst pressure is 10–50% higher for PDMS–PC than PDMS–PMMA bonds, which makes PC a good choice for high-pressure or high flow-rate applications.

### 4.5. PS

Polystyrene (PS) is another optically transparent material that is frequently used material for cell cultures and biosensor applications, thanks to its good biocompatibility [57,94,95]. For PDMS–PS bonding, three previously discussed techniques were also successfully applied, namely, surface activation combined with either APTES [38,40], APTES/TESPSA [46], or APTES + low molecular weight PDMS linker [47] treatment.

A one-step bonding technique, which stands out from the other chemical gluing methods, was also presented by Xu et al. [36]. They used air plasma (a mixture of oxygen and nitrogen) to activate the surface of both PS and PDMS substrates. Oxygen is known to create a large number of hydroxy (–OH) groups with a small portion of carbonyl (–C=O) groups on the surface of carbon-based plastics. The presence of nitrogen in the plasma enables the formation of imine groups ((R1, R2)C=NH) and their free radical forms, which is the essential contribution for irreversible PDMS–plastic bonding [36,96]. This effect of nitrogen was confirmed by both XPS performed on the treated surfaces and negative controls with oxygen/argon mixed plasma. Although this one-step bonding process was reproduced with COC and PP polymers, and they all performed well at dynamic leakage and burst tests (e.g., burst pressures above 500 kPa, measured with compressed air), this technique was still not tested for other mainstream polymers (PMMA, PC, PET, etc.).

Li et al. used an adhesive-based gluing technique to attach PDMS to PS by subsequently spin coating a silicone-based adhesive (PrimeCoat, ~1 µm) and an epoxy adhesive (3–4 µm) onto the substrates [57]. The resulting maximum burst pressure was comparable to other chemical gluing methods (see Table 2). The same adhesive-based technique was successfully implemented to bond PDMS with glass and PET as well [57].

### 4.6. PET

Poly(ethylene terephthalate) (PET) is optically transparent, strong, and lightweight plastic that is one of the favored substrates to choose when the planned application requires flexibility. Its chemical inertness, good gas permeability, and low cost can also be advantageous for several applications. Most previously mentioned chemical gluing technique can be selected for PDMS–PET bonding (namely, surface activation combined with either MPTMS [43], APTES/TESPSA [46], APTES/GPTMS [44,45], or APTES + low molecular weight PDMS linker [47] treatments. The resulting tensile strengths and burst pressures are a bit mixed (e.g., compared to PDMS–PMMA higher [43] and lower [47] tensile strengths are also reported with the same protocols), but all listed approaches can be successfully used to create reliable bonding with this flexible substrate.

Here, another technique that stands out has to be noted. Baraket et al. used KOH-based activation and hydrolyzed the surface of the tested polymers, which were then functionalized with MPTMS. Subsequently, oxygen plasma was made to substitute the propyl–thiol chain with hydroxyl groups by cleaving the terminal groups on the MPTMS treated polymers. These silanol groups form the siloxane bonds with the surface-activated PDMS [20]. Please note that this proposed approach is different from other chemical gluing methods which use MPTMS, e.g., proposed by Wu et al. [43], where the organosilane is bound to the corona treated surface of the PET substrate through thiol groups. The silane layer is in turn bonded with the activated PDMS, as illustrated in Figure 2d. Although Baraket et al. successfully applied this method to both PI and PEN besides PET [20], since no tensile or burst tests were performed, its comparison with the method of Wu et al. is not directly possible.

### 4.7. PI

Polyimides (including DuPont’s favored commercial product, ‘Kapton’) are popular substrate materials for flexible electronic devices [97,98]. One of their main advantages compared to the similarly flexible PET is their high glass transition temperature and stability over a wide temperature range (e.g., between −269 °C and 400 °C) [53]. Its high dielectric constant, good chemical resistance and compatibility with microelectronics packaging technologies also make it an excellent candidate to bridge the gap between microelectronics and microfluidics. The most detailed investigation of PDMS–PI bonding was performed by Hoang et al., who compared chemical gluing methods with adhesives (epoxy, silicon) and with hybrid MPTMS-epoxy adhesive methods, and tested the resulting bonds with peel tests [53]. Using adhesives only (e.g., LePage Gel epoxy adhesive), the resulting peel strengths were quite low (1.7–72 N/m). Out of the two chemical gluing methods, they only obtained a strong bond with the MPTMS/GPTMS pair (200 N/m), the APTES/GPTMS pair yielded a 100-times smaller 2.7 N/m peel strength. This result is somewhat surprising, since other groups successfully used the APTES/GPTMS pair with similar procedures (functionalizing the PDMS with APTES and the plastic with GPTMS) [21,44]. For PDMS–PI bonding, the best results were obtained by combining MPTMS surface functionalization with the epoxy adhesive (470 N/m). Both the MPTMS-epoxy and the MPTMS/GPTMS strategy yielded peel strengths that are comparable or even better than some values reported for PDMS–PDMS pairs [54,55], which indicates a strong and reliable bond.

### 4.8. Other Polymer Substrates

In Table 2, protocols that can be used for bonding PDMS with five more polymers are also given. These strategies were previously discussed in accordance with other substrates. In short, the air plasma surface activation used by Xu et al. was also successfully applied to bond PDMS with polypropylene (PP) and cyclic olefin copolymer (COC) [36]. For COC, Cortese et al. investigated other protocols from oxygen plasma treatment to oxygen plasma + APTES or GPTMS treatment, including oxygen plasma + APTES/GPTMS combined functionalization [21], which yielded the best results. For polyethylene naphthalate (PEN), the protocol of Baraket et al. was used with success, including KOH surface activation, MPTMS treatment, and oxygen plasma [20]. For polyvinyl chloride (PVC), a simple corona activation combined with MPTMS treatment yielded high bonding strengths [43], comparable to PDMS–PMMA or PDMS–PET with the same protocol. Finally, oxygen plasma combined with APTES was successfully used to bond PDMS with acrylonitrile butadiene styrene (ABS) as well.

### 4.9. Metals

Although metals cannot be considered as typical substrates in microfluidic fabrication, it has to be noted that by simple chemical gluing, PDMS can be conveniently bonded with various metals. The thiol–metal bonds, which were discussed in accordance with gold-coated glass [50], were successfully used to bond PDMS with iron, copper, aluminum, or brass by using a simple surface activation + MPTMS treatment protocol [43].

## 5. Conclusions

Strategies and methods to bond PDMS with various rigid and flexible substrate materials were collected and evaluated. As it was shown, with optimized process parameters, high-quality and high-strength bonds can be achieved. Commonly achievable strengths with optimized process parameters—quantitatively, based on the three most widely used testing methods, which are tensile strength tests, burst tests, and channel leakage tests—are >400 kPa tensile strength, >500 kPa burst pressure, or flow rates equal to several 1000× of the internal volume of the channels per minute. This could be achieved with simple surface activation methods for silicone-based substrates (e.g., glass, silicon, or PDMS). For polymers and metallic surfaces, chemical gluing is advised. In some exceptional cases, a combined adhesive + chemical gluing method could yield extra-strong bonds. It is hoped that the collected optimized process parameters in Table 3 will help others in this field to quickly tune their processes based on their substrates, available equipment, or application area.

Despite the growing trend and need for LoC and Micro-TAS devices, PDMS-based microfluidics remains an academic and small-scale R&D, rather than an industrial/commercial success, mostly owing to the scalability problems related to the micromachining and prototyping technologies considering large scale manufacture [99]. However, as demonstrated in the paper, high-quality bonds can now be formed between PDMS and many other plastic materials, some of which are compatible with either large-scale fabrication methods (e.g., PMMA), or electronics packaging technologies (e.g., PI). The combination of PDMS with such materials might open the door for its larger commercial/industrial success in the near future.

## Figures and Tables

**Table 1:** The most frequently used bonding strength measurement methods and test.

No	Name of the Test	Required Equipment	Measured Value	Complexity	Special Sample Preparation Requirements	Restrictions/Possible Problems	Ref.
1	Manual peeling/ delamination test	Performed by hand	Bond formation (yes/no)	+	-	-Only as a first attempt to verify bond formation. -Results are subjective. -A successful bond might be too strong to peel manually.	[19,20,21,34,35,36,37]
2	Adhesive- assisted tensile strength measurement	Tensile testing machine, adhesive	Tensile strength [Pa]	+	An adhesive must be applied to the samples	-The tensile strength of the adhesive bond must be higher than the quantified bond between PDMS and substrate.	[38]
3	Cylinder-based tensile strength measurement	Tensile testing machine	Tensile strength [Pa]	++	Requires a special molding form for the PDMS.	-The size of the cylinder should be optimized to avoid rupture, and thin substrates are vulnerable to breaking. -Due to the special PDMS geometry, it cannot be used on functional elements or combined with other test methods.	[39,40]
4	Double- substrate-bonding-based tensile strength measurement	Tensile testing machine	Tensile strength [Pa]	++	Two substrates are required to be bonded to the PDMS. If screws are used to fix the sample to the machine, holes must be drilled into the substrate.	-The areas of the two bonds should be precisely controlled, the tensile strength of the bond should be calculated accordingly.	[41,42]
5	String-based tensile strength measurement (Pull test)	Tensile testing machine	Tensile strength [Pa]	+++	The PDMS block and a string should be cured together. A drilled hole is needed in the substrate.	-The bond between the string and PDMS is crucial. The string might be torn out from the PDMS block without detaching the PDMS from the substrate. -The obtained tensile strength might not be directly comparable with other methods.	[43,44,45,46,47]
6	Shear strength measurement	Shear testing machine	Shear strength [Pa](shear strain)	++	The width of the PDMS block should not be wider than the peeling tool.	-Parallel forces should be maintained, PDMS bending is a problem. -The obtained shear strength should be treated with reservations.	[48,49]
7	Lap shear strength measurement	Tensile testing machine	Lap shear strength [Pa]	++	Either two PDMS blocks are bonded to one substrate or two substrates to one PDMS block.	-The sizes of the bonded areas are crucial and should be properly controlled. -The shear strength result has to be calculated accordingly. -The clamping of the PDMS blocks might be difficult.	[50]
8	Peel test	Tensile testing machine, glass slide, adhesive	Peel strength [N/m]	+++	A partially bonded flexible foil, is required. The PDMS is bonded to a fixed support (e.g., a glass slide) with adhesive.	-The pulling angle should be kept 90° during the experiment to ensure normal forces. The movement of the supporting glass slide should be controlled accordingly. -The strength of the adhesive bond should be higher.	[53]
9	T-peel test	Tensile testing machine	Peel strength [N/m]	++	A partially bonded formation is required. A thin foil or a second PDMS substrate can be used.	The forces acting on the bond should be kept normal.	[54,55]
10	Static leakage test (durability test)	Ink, tubes, syringe, microfluidic cell	Time until leakage [h]	+	Microfluidic channels are required inside the PDMS block	-Proper ink leakage detection. -Might be time-consuming for good-quality bonds.	[20,40,42]
11	Dynamic leakage test	Ink, tubes, syringe, microfluidic cell, computer -controlled pump	Max. flow rate before leakage [mL/min]	++	Microfluidic channels are required inside the PDMS block.	Proper ink leakage detection.	[19,35,36,38,43,44,45,46,47,50,56,57]
12	Burst test	Compressed air or fluid, microfluidic cell, syringe pump, high-pressure tank, pressure sensor	Burst pressure [Pa]	++	A specific blister should be formed in the PDMS block.	-The size of the blister is important and should be properly controlled. -PDMS might explode from the substrate at high pressures.	[21,36,37,38,39,40,41,46,47,48,57,59,60,62]

**Table 2:** Bond strengths measured with different test methods on PDMS–substrate bonds, categorized by the substrate type. In the table only the test results corresponding to the optimized process parameters are given.

Substrate Type	Bonding Technology (see Table 3)	Test Method (see Table 1)	Test Results		Ref.
			Value	Parameter/Condition	
PDMS	Corona treatment	11	>60 µL/min	max. tested flow rate, ~1500 × min^−1^ internal volume	[19]
		12	290 kPa	average burst pressure (air), variation: 227–380 kPa	[60]
	O_2_ plasma	5	91 kPa	tensile strength	[45]
		9	190 N/m	peel strength, for optimized plasma power	[55]
		11	10 mL/min	max. flow rate, ~30,000 × min^−1^ internal volume	[56]
		12	300 kPa	average burst pressure (air), variation: 180–515 kPa	[60]
		12	400 kPa	burst pressure (N_2_), with optimized parameters (range: 69–400 kPa)	[59]
		12	105–180 kPa	burst pressure (air)	[62]
	Air plasma	9	400 N/m	max. peel strength, with optimized parameters	[54]
	O_2_ plasma + APTES / GPTMS surface treatment	5	184 kPa	tensile strength	[45]
		11	5 mL/min	no leakage was observed, 2000 × min^−1^ internal volume	[45]
	UV illumination	12	87–95 kPa	burst pressure (air)	[62]
	DMPMS pre- polymer gluing	11	330–500 µL/min	max. flow rate, ~6600 × min^−1^, depending on the preparation	[35]
	PDMS pre- polymer gluing	12	671 kPa	average burst pressure (air), variation: 545–690 kPa	[60]
		12	>500 kPa	burst pressure (air)	[62]
Glass	Corona discharge	7	94 kPa	max. lap shear strength	[50]
	O_2_ plasma	6	125–245 kPa	max. shear stress, for bonding areas between 10–90 mm^2^	[48]
		6	70–113 kPa	max. shear stress	[49]
		11	4 mL/min	max. flow rate before leakage ~12000 × min^−1^ internal volume	[56]
		12	185–270 kPa	burst pressure (air)	[62]
		12	190–610 kPa	burst pressure (ink), for bonding areas between 10–90 mm^2^	[48]
		12	510 kPa	burst pressure (N_2_), with optimized parameters (range: 276–510 kPa)	[59]
	UV illumination	12	270–314 kPa	burst pressure (air)	[62]
	DMPMS pre- polymer gluing	11	500 µL/min	max. flow rate, ~6600× min^−1^, with optimized preparation	[35]
	PDMS pre- polymer gluing	12	>500 kPa	burst pressure (air)	[62]
Parylene coated silicon	PDMS pre- polymer gluing	3	400 kPa	tensile strength	[39]
		12	36 kPa	burst pressure (water)	[39]
	Plasma treatment with N_2_ and SF_6_ mixture	3	1.4 MPa	bond tensile strength with optimized parameters	[39]
		12	145 kPa	burst pressure (water)	[39]
Gold-coated glass	UV illumination + MPTMS surface treatment	7	120 kPa	max. lap shear strength	[50]
PMMA	PDMS pre- polymer gluing	4	15 kPa	tensile strength	[42]
	Corona discharge	5	336 kPa	tensile strength	[43]
	Corona discharge + APTES surface treatment + epoxy term. PDMS linker	5	306 kPa	tensile strength	[47]
		11	45 mL/min	no leakage for 3000× min^−1^ internal volume	[47]
		12	586 kPa	burst pressure (air)	[47]
	O_2_ plasma + APTES surface treatment	2	385 kPa	tensile strength	[38]
		4	1.6 MPa	max. tensile strength, with optimized APTES functionalization	[41]
		11	60 mL/min	no leakage at 24,000 × min^−1^ internal volume	[38]
		12	100 kPa	delamination pressure in water, >500 kPa in air	[37]
		12	260 kPa	average burst pressure (water), with optimized APTES functionalization	[41]
	O_2_ plasma + APTES surface treatment + Corona discharge	4	2.5 MPa	max. tensile strength, with optimized APTES functionalization	[41]
		12	300 kPa	average burst pressure (water), with optimized APTES functionalization	[41]
	O_2_ plasma + APTES/TESPSA surface treatment	5	259 kPa	tensile strength	[46]
		11	30 mL/min	no leakage at 3000× min^−1^ internal volume	[46]
		12	345 kPa	burst pressure (air)	[46]
	O_2_ plasma + APTES/GPTMS surface treatment	5	180 kPa	tensile strength	[44]
		11	30 mL/min	no leakage at 2000× min^−1^ internal volume	[44]
		12	510–538 kPa	burst pressure (air)	[44]
PC	Corona discharge + MPTMS surface treatment	5	511 kPa	tensile strength	[43]
	Corona discharge + APTES surface treatment + epoxy terminated PDMS linker	5	220 kPa	tensile strength	[47]
		12	620 kPa	burst pressure (air)	[47]
	O_2_ plasma + APTES surface treatment	2	430–490 kPa	tensile strength	[38]
		11	60 mL/min	no leakage at 24,000 × min^−1^ internal volume	[38]
		12	>228 kPa	burst pressure (water)	[58]
		12	100 kPa	delamination pressure in water, >500 kPa in air	[37]
	O_2_ plasma + APTES/TESPSA surface treatment	5	477 kPa	tensile strength	[46]
		11	30 mL/min	no leakage at 3000× min^−1^ internal volume	[46]
		12	413 kPa	burst pressure (air)	[46]
	O_2_ plasma + APTES / GPTMS surface treatment	5	178 kPa	tensile strength	[44]
		11	30 mL/min	no leakage at 2000× min^−1^ internal volume	[44]
		12	524–579 kPa	burst pressure (air)	[44]
PS	Corona discharge + APTES surface treatment + epoxy terminated PDMS linker	5	476 kPa	tensile strength	[47]
		12	620 kPa	burst pressure (air)	[47]
	Air plasma	10	-	no leakage with 1 M HCl for 3 days, 1 M NaOH for 1 week, water for 1 month	[36]
		11	15 mL/min	no leakage at 9000× min^−1^ internal volume	[36]
		12	>500 kPa	burst pressure (air)	[36]
	O_2_ plasma + APTES surface treatment	2	388 kPa	tensile strength	[38]
		11	60 mL/min	no leakage at 24,000 × min^−1^ internal volume	[38]
		3	12 kPa	tensile strength	[40]
	O_2_ plasma + APTES / TESPSA surface treatment	5	520 kPa	tensile strength	[46]
		11	30 mL/min	no leakage at 3000× min^−1^ internal volume	[46]
		12	448 kPa	burst pressure (air)	[46]
	O_2_ plasma + epoxy adhesive	11	500 µL/min	no leakage at 1000× min^−1^ internal volume	[57]
		12	>414 kPa	burst pressure (air)	[57]
PET	Corona discharge + APTES surface treatment + epoxy terminated PDMS linker	5	189 kPa	tensile strength	[47]
	Corona discharge + MPTMS surface treatment	5	476 kPa	tensile strength	[43]
	O_2_ plasma + APTES / GPTMS surface treatment	1	-	successful bonding	[45]
		12	579 kPa	burst pressure (air)	[44]
	O_2_ plasma + APTES/TESPSA surface treatment	5	458 kPa	tensile strength	[46]
		11	30 mL/min	no leakage at 3000× min^−1^ internal volume	[46]
		12	379 kPa	burst pressure (air)	[46]
	KOH activation + MPTMS surface treatment + O_2_ plasma	1	-	successful bonding— no delamination with optimized parameters	[20]
		10	-	no leakage during 1 month storage	[20]
PI	epoxy adhesive	8	1.7 N/m	peel strength	[53]
	UV/ozone + silicone adhesive	8	72 N/m	peel strength	[53]
	UV/ozone treatment + APTES/GPTMS surface treatment	8	2.7 N/m	peel strength	[53]
	UV/ozone treatment + MPTMS/GPTMS surface treatment	8	200 N/m	peel strength	[53]
	UV/ozone treatment + MPTMS by liquid deposition + epoxy adhesive	8	470 N/m	peel strength	[53]
	KOH activation + MPTMS surface treatment + O_2_ plasma	1	-	successful bonding— no delamination with optimized parameters	[20]
		10	-	no leakage for 1-month storage	[20]
PP	Air plasma	10	-	no leakage with 1 M HCl for 3 days, 1 M NaOH for 1 week, water for 1 month	[36]
		11	15 mL/min	no leakage at 9000× min^−1^ internal volume	[36]
		12	>500 kPa	burst pressure (air)	[36]
COC	Air plasma	12	>500 kPa	>500 kPa burst pressure (air)	[36]
		11	15 mL/min	no leakage at 9000× min^−1^ internal volume	[36]
		10	-	no leakage with 1 M HCl for 3 days, 1 M NaOH for 1 week, water for 1 month	[36]
	O_2_ plasma	1	-	the bond was not permanent	[21]
		12	150 kPa	burst pressure (air)	[21]
	O_2_ plasma + APTES surface treatment	1	-	strong bond	[21]
		12	380 kPa	burst pressure (air)	[21]
		2	432 kPa	tensile strength	[38]
		11	60 mL/min	no leakage at 24,000 × min^−1^ internal volume	[38]
	O_2_ plasma + GPTMS surface treatment	1	-	strong bond	[21]
	O_2_ plasma + APTES/GPTMS surface treatment	1	-	stronger bond than with only APTES or GPTMS	[21]
		12	>800 kPa	burst pressure (air), after 6 months ≥ 700 kPa	[21]
ABS	O_2_ plasma + APTES surface treatment	12	100 kPa	delamination pressure in water, >500 kPa in air	[37]
PEN	KOH activation + MPTMS surface treatment + O_2_ plasma	1	-	successful bonding— no delamination with optimized parameters	[20]
		10	-	no leakage for 1-month storage	[20]
PVC	Corona discharge + MPTMS surface treatment	5	467 kPa	tensile strength	[43]

**Table 3:** Optimized process parameters for bonding PDMS with different substrates. For the bonding strength test results see Table 2. Abbreviations: APTES, 3-aminopropyl)triethoxysilane; CNC, Computer Numerical Control; COC, Cyclic Olefin Copolymer; DMPMS, Dimethyl-MethylPhenylMethoxy Siloxane; DRIE, Deep Reactive Ion Etching; GPTES, 3-glycidyloxypropyl)triethoxysilane; GPTMS, 3-glycidyloxypropyl)trimethoxysilane; KOH, Potassium hydroxide; MPTMS, 3-mercaptopropyl)trimethoxysilane; NOA74, Norland Optical Adhesive; PC, Polycarbonate; PDMS, Polydimethylsiloxane; PEN, Polyethylene Naphthalate; PET, Polyethylene Terephthalate; PI, Polyimide; PMMA, Poly(methyl methacrylate); PS, Polystyrene; PVC, Polyvinyl Chloride; RT, Room Temperature; TESPSA, 3-triethoxysilyl)propylsuccinic anhydride; UV, Ultraviolet; Physical quantities: R–PDMS curing agent/base polymer ratio, t—time, P—RF plasma power, p—pressure, f—gas flow rate. Conc.—concentration. The label in the bracket refers to the used medium as a—aqueous, e—ethanol, m—methanol.

Substrate Type	Molding Form and Fabrication Technology	PDMS Curing			Surface Activation Parameters					Chemical Gluing Parameters				Adhesives	Treated Side		Bonding Conditions			Ref.
		*R* [[–]	*T* [°C]	*t* [min]	Type	*t* [s]	*P* [W]	*p* [mTorr]	*f* [sccm]	Type	Conc. [%]	*T* [°C]	*t* [min]		PDMS	Substrate	*T* [°C]	*t* [min]	*p* [MPa]	
PDMS	NA	1:10	60	60	corona	30–40	-	-	-	-	-	-	-	-	activation	activation	RT	30–60	-	[19]
PEN, PET, PI	PDMS: one-step photolithography and deep reactive ion etching (DRIE) plastics: -	1:10	90	60	O_2_ plasma (PDMS)3M KOH activation + O_2_ plasma (plastics)	20 10	0.4/cm^2^ 0.4/cm^2^	100 100	-	MPTMS	5.5 (m)	RT	120	-	activation	KOH activation + MPTMS + plasma treatment	-	-	-	[20]
COC	PDMS: -substrate: hot embossing	1:10	80	60	O_2_ plasma	20	500	8	-	APTES GPTMS	5 (a) 5 (a)	50 50	60 60	-	activation +GPTMS	activation +APTES	80	120	-	[21]
PDMS, glass	PDMS: SU-8 soft-lithography substrate: -	1:10	75	60	-	-	-	-	-	-	-	-	-	pre-polymer gluing with DMPMS (7 µm, spin coated)	pre-polymer glue	pre-polymer glue	70	240	0.02	[35]
COC, PP, PS	PDMS: soft-lithography, glass mold fabricated by microlithography plastic: -	1:10	80	60	air plasma	30	150 (COC) 250 (PP) 150 (PS)	75	14	-	-	-	-	-	activation	activation	-	-	-	[36]
ABS, PC, PMMA	PDMS: one-step photolithography and deep reactive ion etching (DRIE) plastics: -	1:10	60	60	O_2_ plasma	60–300	50	1500	-	APTES	5 (m)	60	20	-	activation	activation + APTES	80	60	-	[37]
COC, PC, PMMA, PS	PDMS: SU-8 soft-lithography plastics: -	1:10	80	30	O_2_ plasma	60	60	-	-	APTES	1 (a)	RT	20	-	activation + APTES	activation	RT	60	-	[38]
parylene C on glass	PDMS: SU-8 soft-lithography substrate: -	1:10	RT	720	mixed plasma	60	300	5	50 (N_2_) 30 (SF_6_)	-	-	-	-	pre-polymer gluing with PDMS	pre-polymer glue + plasma treatment	pre-polymer glue + plasma treatment	RT	720	-	[39]
PS	PDMS: SU-8 soft-lithography plastic: -	1:13	65	120	O_2_ plasma	60	30	320	-	APTES	1 (a)	RT	20	-	activation	activation + APTES	65	60	-	[40]
PMMA	PDMS: SU-8 soft-lithography plastic: -	1:10	65	240	O_2_ plasma corona	60 60	200 -	- -	50 -	APTES	5 (a)	85	1	-	activation (corona)	activation (O_2_) + APTES + corona	65	120	-	[41]
PET, PI, PP, PS, PVC, metals	PDMS: -substrates: -	-	-	-	corona	120	-	-	-	MPTMS	2 (a)	RT	1-5	-	activation	MPTMS + corona treatment	RT	10	-	[43]
PC, PET, PI, PMMA	PDMS: SU-8 soft-lithography plastics: hot embossing	1:10	80	30	O_2_ plasma	60	50-60	-	-	APTES GPTES	1 (a) 1 (a)	RT RT	20 20	-	activation + APTES	activation + GPTES	RT	60	-	[44]
PDMS, PET	PDMS: SU-8 soft-lithography plastic: -	1:10	80	30	O_2_ plasma	60	-	-	-	APTES GPTMS	1 (a) 1 (a)	RT RT	20 20	-	activation + APTES	activation + GPTMS	RT	60	-	[45]
PC, PET, PMMA, PS	PDMS: -plastics: CNS milling, engraving	1:10	80	120	O_2_ plasma	60	-	-	-	APTES TESPSA	1 (a) 1 (a)	RT RT	30 30	-	activation + TESPSA	activation + APTES	RT	-	-	[46]
PC, PET, PMMA, PS	PDMS: SU-8 soft-lithography plastics: CNC milling, engraving	1:10	80	60	corona	60	-	-	-	APTES	5 (a)	80	20	monoglycidyl ether terminated, low-molecular-weight PDMS was added at 80 °C for 4 h	activation	activation + APTES + adhesive	25	15	0.1	[47]
glass	PDMS: SU-8 soft-lithography substrate: -	1:10	60 + 100	45 + 135	O_2_ plasma	35 (PDMS) 120 (glass)	10.5 (PDMS) 18 (glass)	- -	60 (glass) 40 (PDMS)	-	-	-	-	-	activation	activation	-	-	-	[48]
glass	-	1:10	100	60	O_2_ plasma	300	200	-	60	-	-	-	-	-	activation	activation	75	300	0.5	[49]
gold on glass	PDMS: SU-8 soft-lithography substrate: -	1:10	70	60	UV/ozone	300	-	-	-	MPTMS	2 (e)	RT	60	-	activation + MPTMS	cleaning	60	60	-	[50]
glass	PDMS: SU-8 soft-lithography substrate: -	1:10	70	60	corona	120	-	-	-	-	-	-	-	-	activation	activation	80	720	-	[50]
PI	-	1:10	60	120	UV/ozone	600	-	-	-	MPTMS GPTMS	1 (m) 1 (m)	RT RT	60 60	epoxy adhesive (LePage Gel epoxy adhesive) —optionally	activation + MPTMS	activation + GPTMS or MPTMS + epoxy	RT	720	0.03	[53]
PDMS	-	1:10	200	8	air plasma	50	18	200	-	-	-	-	-	-	activation	activation	RT	5	0.03	[54]
PDMS	-	1:10	80	60	O_2_ plasma	300	300	atm	15	-	-	-	-	-	activation	activation	160	20	1.4	[55]
PDMS, glass	PDMS: SU-8 soft-lithography substrate: -	1:10	65	60	O_2_ plasma	12	150	-	-	-	-	-	-	-	activation	activation	-	-	-	[56]
PS	PDMS: SU-8 soft-lithography plastic: -	1:10	60	180	O_2_ plasma	30 (PDMS) 75 (PS)	18	45	100	-	-	-	-	silicone adhesive (PrimeCoat, 1 µm) epoxy adhesive (Epoxy 301-2, 3-4 µm)	activation + adhesives	activation	60	180	-	[57]
glass	PDMS: SU-8 soft-lithography	-	-	-	O_2_ plasma	20	20	1000	-	-	-	-	-	-	activation	activation	-	-	-	[59]
PDMS	mold fabricated by Xurography	1:10	60	720	corona	30	-	-	-	-	-	-	-	-	activation	activation	-	-	-	[60]
PDMS	mold fabricated by Xurography	1:10	60	720	O_2_ plasma	20	20	700	-	-	-	-	-	-	activation	activation	-	-	-	[60]
COC, PET, PMMA, PS, glass, metals	PDMS: SU-8 soft-lithography plastics: -	1:10	80	90	O_2_ plasma	60	25	-	-	APTES	1 (a)	RT	20	epoxy adhesive (NOA74), deposited by spin coating (6000 rpm 1 min), cured with UV lamp (20 mW/cm^2^)	activation + APTES + NOA74 epoxy glue	-	-	90	-	[61]
PDMS, glass	PDMS: Aluminium mold, CNC milled substrate: -	1:10	70	180	O_2_ plasma UV/ozone	180–300 180–300	- -	- -	-	-	-	-	-	-	activation	activation	80	15	-	[62]

## Data Availability

Not applicable.

## Abbreviations

APTES: 3-aminopropyl)triethoxysilane; CNC, Computer Numerical Control; COC, Cyclic Olefin Copolymer; DMPMS, Dimethyl-MethylPhenylMethoxy Siloxane; DRIE, Deep Reactive Ion Etching; GPTES, 3-glycidyloxypropyl)triethoxysilane; GPTMS, 3-glycidyloxypropyl)trimethoxysilane; ICP, Inductively Coupled High-Density Plasma System; KOH, Potassium hydroxide; LoC, Lab-on-a-Chip; MEMS, Micro Electro-Mechanical Systems; micro-TAS, micro Total Analysis Systems; MPTMS, 3-mercaptopropyl)trimethoxysilane; NOA74, Norland Optical Adhesive; PC, Polycarbonate; PDMS, Polydimethylsiloxane; PECVD, Plasma Enhanced Chemical Vapor Deposition; PEN, Polyethylene Naphthalate; PET, Polyethylene Terephthalate; PI, Polyimide; PMMA, Poly(methyl methacrylate); PS, Polystyrene; PVC, Polyvinyl Chloride; R&D, Research and Development; RIE, Reacting Ion Etching; RT, Room Temperature; SPR, Surface Plasmon Resonance; TESPSA, 3-triethoxysilyl)propylsuccinic anhydride; THF, Tetrahydrofuran; UV, Ultraviolet.
